# The Tumor Cytosol miRNAs, Fluid miRNAs, and Exosome miRNAs in Lung Cancer

**DOI:** 10.3389/fonc.2014.00357

**Published:** 2015-01-05

**Authors:** Xin Qin, Haisheng Xu, Wenrong Gong, Wenbin Deng

**Affiliations:** ^1^Medical College, Hubei University of Arts and Science, Xiangyang, China; ^2^Department of Oncology, Xiangyang Central Hospital, Xiangyang, China; ^3^Department of Biochemistry and Molecular Medicine, University of California Davis School of Medicine, Sacramento, CA, USA

**Keywords:** microRNA, biomarker, lung cancer

## Abstract

The focus of this review is to provide an update on the progress of microRNAs (miRNAs) as potential biomarkers for lung cancer. miRNAs are single-stranded, small non-coding RNAs that regulate gene expression and show tissue-specific signatures. Accumulating evidence indicates that miRNA expression patterns represent the *in vivo* status in physiology and disease. Moreover, miRNAs are stable in serum and other clinically convenient and available tissue sources, so they are being developed as biomarkers for cancer and other diseases. Cancer is currently the primary driver of the field, but miRNA biomarkers are being developed for many other diseases such as cardiovascular and central nervous system diseases. Here, we examine the framework and scope of the miRNA landscape as it specifically relates to the translation of miRNA expression patterns/signatures into biomarkers for developing diagnostics for lung cancer. We focus on examining tumor cytosol miRNAs, fluid miRNAs, and exosome miRNAs in lung cancer, the connections among these miRNAs, and the potential of miRNA biomarkers for the development of diagnostics. In lung cancer, miRNAs have been studied in both cell populations and in the circulation. However, a major challenge is to develop biomarkers to monitor cancer development and to identify circulating miRNAs that are linked to cancer stage. Importantly, the fact that miRNAs can be successfully harvested from biological fluids allows for the development of biofluid biopsies, in which miRNAs as circulating biomarkers can be captured and analyzed *ex vivo*. Our hope is that these minimally invasive entities provide a window to the *in vivo* milieu of the patients without the need for costly, complex invasive procedures, rapidly moving miRNAs from research to the clinic.

## Introduction

Lung cancer is the most common type of cancer worldwide. It is estimated that there are 430,090 men and women living in the United States with a history of lung cancer, and that an additional 224,210 cases will be diagnosed in 2014 (1). Lung cancer is also the leading cause of cancer-related death (2). Because of the lack of validated population-based screening procedures, most patients with lung cancer are diagnosed at an advanced stage. Consequently, the overall 5-year survival rate is only 15% (3). Therefore, there is an urgent need to identify reliable biomarkers of lung cancer, which can then be used for improving accuracy of diagnosis, predicting prognosis, and monitoring disease progression and response to therapy.

MicroRNAs (miRNAs) are small, non-coding RNAs, 19–24 nucleotides in length. They negatively regulate the expression of multiple genes either by inducing translational silencing or by causing the degradation of messenger RNAs (mRNAs) of the targeted gene, both via incomplete base-pairing to a complementary sequence in the 3′-untranslated region (UTR) (4). Since the discovery of the first miRNA, lin-4, in *Caenorhabditis elegans* (5), more than 1,800 human precursor miRNAs have been characterized (6). The accumulating data indicate that miRNAs play important roles in tumorigenesis, metastasis, and drug responsiveness in lung cancer, and can be potential biomarkers for lung cancer (7, 8). Current research has found that the miRNAs can not only be detected in tumor tissues but also in body fluids and even in some extracellular organelles, such as exosomes, all of which have the potential to serve as biomarkers for lung cancer. In this article, we summarize the progress on miRNAs originating from three different sources (tumor tissues, body fluids, and exosomes) as biomarkers for lung cancer.

## The Tumor Cytosol miRNAs, Fluid miRNAs, and Exosome miRNAs in Lung Cancer

### The tumor cytosol miRNAs in lung cancer

Many factors, including variations of chromatin, epigenetic factors, hypoxia, and changes in hormone levels, can affect the expression profiles of tumor cytosolic miRNAs. Differences between miRNAs in tumor tissues and normal tissues have been studied extensively and profoundly, and data collected from these studies indicate that miRNAs are involved in several critical processes of lung cancer including the initiation, metastasis, and drug response.

In 2004, Takamizawa et al. (9) identified the first miRNA family, let-7, which was associated with the tumorigenesis of lung cancer. In their study, they found that introduction of let-7a and let-7f isoforms into A549 cells, a lung adenocarcinoma cell line with low baseline levels of let-7 expression, significantly inhibited the growth of A549 cells. This was further validated clinically, where significantly shorter patient survival after diagnosis was associated with reduced *let-7* expression. Subsequently, many targets of let-7 have been identified, including the RAS family (10), HMGA2 (10–12), c-Myc (13, 14), CDC25A, CDK6, and Cyclin D2 (15), which elucidated the mechanisms by which let-7 exerts its function in tumorigenesis. Since then, many miRNAs were identified as oncogenes or tumor suppressor genes, such as miR-17–92 (16, 17), miR-218 (18), miR-21 (19), and miR-34 family (miR-34a and miR-34b/c) (20–24).

MicroRNAs not only play pivotal role in tumorigenesis of lung cancer but also are involved in tumor metastasis. Several miRNAs including miR-17–92 (25–28), miR-200 family of miRNAs (miR-200a, miR-200b, miR-200c, miR-141, and miR-429) (29), miR-125a-3p/5p (30), miR-21 (31), and miR-106b-25 cluster (miR-106b and miR-93) (32) are reported to be related to the metastasis of lung cancer.

MicroRNAs are also involved in the drug responsiveness of lung cancer cells. It was reported that overexpression of miR-181b could sensitize A549/Cisplatin (CDDP) cells to CDDP-induced apoptosis by decreasing the levels of the anti-apoptotic protein BCL2 (33). Additionally, miR-181a and miR-630 were reported to be modulators of CDDP response in non-small-cell lung cancer (NSCLC) A549 cells (34). In contrast, down-regulation of miR-17-5p expression was associated with paclitaxel resistance by up-regulation of the autophagic protein Beclin 1 (BECN1) expression in NSCLC (35). Similarly, let-7a, miR-126, and miR-145 could sensitize the responsiveness of the large-cell cancer cell line H460 and A549 cells to Gefitinib (36).

### Body fluid miRNAs in lung cancer

In addition to tumor tissues, miRNAs are also found in body fluids such as blood, serum, plasma, urine, and cerebrospinal fluid (CSF), as well as in sputum, saliva, and bronchoalveolar lavage (BAL) (37, 38). Several studies indicate that body fluid miRNAs are stable even under extreme conditions, such as repeated freeze-thaw cycles and extreme pHs (e.g., pH = 1 or pH = 13). This feature makes body fluid miRNAs suitable biomarkers for clinical detection (39).

Chen et al. showed that there is a distinct difference between the profile of miRNAs found in sera of healthy individuals and NSCLC patients. Compared to healthy sera, the expressions of 28 different miRNAs were down-regulated and 63 different miRNAs were up-regulated in lung cancer patients. The expression levels of miRNA-25 and miRNA-223, which exhibited the most robust difference in the profile, were further studied in the sera from 152 lung cancer patients and 75 healthy subjects, showing that both of the miRNAs were indeed highly expressed in cancer patient sera. These results indicated that miRNA-25 and miRNA-223 could be used as potential diagnosis biomarkers for NSCLC (40).

Several other miRNAs, including miR-141, miR-155, miR-1254, and miR-574-5p, were identified as potential early diagnostic biomarkers (41, 42). A recent meta-analysis indicated that the early diagnostic value of circulating miR-21 is much better than the plasma miR-21 (43). Roth et al. found that circulating levels of miR-361-3p and miR-625* could be used as blood-based markers for differentiating malignant lung tumors from benign lung tumors (44).

Body fluid miRNAs, especially the circulating miRNAs, can also be promising biomarkers for metastasis and survival time indication. Roth et al. found that high expression levels of miRNA-10b were highly associated with positive lymph node metastasis in lung cancer (41). Hu et al. investigated the circulating miRNA in 303 lung cancer patients and found that the concentrations of 11 miRNAs were elevated more than fivefolds in patients with shorter survival times compared to those whom survived significantly longer. In addition, miR-486, miR-30d, miR-1, and miR-499 were identified as disease fingerprints to predict overall survival in those patients (45). Boeri et al. showed that plasma levels of miR-155, miR-197, and miR-182 could serve as non-invasive biomarkers for early detection and diagnosis of lung cancer. These miRNAs were shown to be significantly elevated in the plasma of the lung cancer patients compared to the cancer free control subjects by greater than 10-folds, and could help discriminate the two groups (46).

### Exosomal miRNAs and lung cancer

Exosomal miRNAs, strictly speaking, are also body fluid miRNAs. However, in contrast to the miRNAs circulating freely in the body fluid, exosomal miRNAs are encapsulated in the cell organelles called the exosomes, which are small (30–90 nm) extracellular vesicles derived from the multivesicular body (MVB) sorting pathway (47). Numerous studies indicate that the expression of miRNAs in exosomes is different in the normal condition and in pathological conditions such as tumor.

Riccardo and colleagues screened 742 miRNAs in circulating exosomes and selected 4 miRNAs (miR-378a, miR-379, miR-139-5p, and miR-200b-5p) as screening markers for segregating lung adenocarcinoma and carcinomas patients from healthy former smokers. They also identified six miRNAs (miR-151a-5p, miR-30a-3p, miR-200b-5p, miR-629, miR-100, and miR-154-3p) for segregating lung adenocarcinoma patients and lung granuloma patients (48).

Guilherme et al. compare 12 specific miRNAs (miR-17-3p, miR-21, miR-106a, miR-146, miR-155, miR-191, miR-192, miR-203, miR-205, miR-210, miR-212, and miR-214) in peripheral circulation exosome-derived miRNAs and tumor-derived miRNAs in lung cancer patients and healthy people. The results showed that there was no significant difference between peripheral circulation miRNA-derived exosomes and miRNA-derived tumors, and thus the exosome-derived miRNAs can be used as biomarkers for lung cancer (49).

Clearly, a number of specific miRNAs or their families show clinical associations in lung cancer and potential values in cancer stages in clinic (Table 1).

**Table 1:** **Selected microRNA signatures in lung cancer and their potential value in clinic**.

miRNA	Location	Signature	Potential value in clinic	Reference
let-7	Tumor tissues	↓	Diagnosis marker	(7)
miR-17-92		↑	Diagnosis marker	(14, 15)
miR-218		↓	Diagnosis marker	(16)
miR-21		↑	Diagnosis marker and metastasis marker	(17, 29)
miR-34a miR-34b/c		↓	Diagnosis marker	(18–22)
miR-200 family		↑	Metastasis marker	(27)
miR-125a-3p/5p		↓	Pathological stage indicator and metastasis marker	(28)
miR-106b-25 cluster		↑	Diagnosis marker	(30)
miR-181b		↑	Predictor for drug resistance to Cisplatin	(31, 32)
miR-181a	
miR-630	
miR-17-5p		↑	Predictor for drug resistance to paclitaxel	(33)
miR-145		↑	Predictor for drug resistance to Gefitinib	(34)

miR-25	Body fluids	↑	Early diagnostic marker	(38)
miR-223		↑	Early diagnostic marker	(38)
miR-141		↑	Early diagnostic marker	(39)
miR-155		↑	Early diagnostic marker	(40)
miR-1254		↑	Early diagnostic marker	(41)
miR-574-5p		↑	
miR-361-3p		↑	Indicator for malignant lung tumors vs benign lung tumors	(42)
miR-625*		↑	
miRNA-10b		↑	Indicator for positive lymph node metastasis	(43)
miR-486		↑	Predictor for overall survival	(44)
miR-30d	
miR-1	
miR-499	
miR-197		↑	Diagnosis marker for lung cancer patients vs normal people	(41)
miR-182	

miR-378a	Exosomes	↑	Diagnosis marker for lung adenocarcinoma and carcinomas patients vs healthy former smokers	(46)
miR-379	
miR-139-5p	
miR-200b-5p	
miR-151a-5p		↑	Diagnosis marker for the lung adenocarcinoma patients vs lung granuloma patients	(46)
miR-30a-3p	
miR-200b-5p	
miR-629		↑	Early diagnostic marker	(47)
miR-100	
miR-154-3p	

## The Isolation and Detection of Tumor Cytosol miRNAs, Fluid miRNAs, and Exosome miRNAs

Tumor cytosol miRNAs can be isolated from fresh tumor tissues or stored formalin-fixed paraffin embedded (FFPE) tissues with Trizol or the conventional phenol/chloroform extraction.

Compared to miRNAs isolated from tumor tissues, the isolation of miRNAs from body fluids requires careful handling in order to avoid the contaminations of proteins in the body fluids. Chen et al. used phenol/chloroform to remove the serum proteins after using the Trizol for isolating miRNAs from serum (40). Researchers have also added proteinase K to the body fluids during extraction of the miRNAs (50). There are also commercially available kits for isolation the body fluid miRNAs such as the PARIs Kit and PAXGene Blood miRNA Kit (Qiagen). The conventional detection method is also applicable for body fluid miRNAs (51).

For detection of exosomal miRNAs, exosomes have to be harvested. The commonly used methods for isolation of exosomes include ultracentrifugation (52) and polymer-based Exoquick reagent (System Bioscience, Inc.) (53). The exosomes can be identified by Western-blot analysis with the two commonly used exosomal markers, the tetraspanin molecule CD63 and tumor susceptibility gene TSG 101 (54).

The actual detection of tumor cytosol miRNAs, fluid miRNAs, and exosome miRNAs use similar strategies such as Northern blot, RT-PCR, miRNA array, or next generation sequence (NGS) (55).

Although the procedures for isolation of miRNAs from body fluids or exosomes are relatively more complex than isolation of miRNAs from tumor tissues, miRNAs from body fluids or exosomes are considered as better biomarkers for lung cancers because they involve non-invasive procedures compared biopsies for extracting tumor-derived miRNAs. Another advantage of using miRNAs from body fluids or exosomes as biomarkers in lung cancer is that once potential biomarkers are identified, methods can be optimized to detect these specific miRNAs for use in larger studies and every-day practice (Table 2).

**Table 2:** **The isolation and detection of tumor cytosol, body fluid and exosome miRNAs**.

Classification	Tumor cytosol miRNAs	Body fluid miRNAs	Exosome miRNAs
Sources	Fresh tumor tissues or FFPE tissues	Whole blood, serum, plasma, urine, CSF, sputum, saliva, or BAL	
Special sample process for miRNA isolation	None	Need to avoid protein contamination	Need to isolate exosomes
Complexity of isolation	+	++	+++
Detection	qRT-PCR, Northern blot, NGS, microarray		
Invasiveness	Yes	None	None
Main function	Diagnosis, prognosis and therapy	Early diagnosis and prognosis	Early diagnosis, prognosis and drug transportation

*FFPE, formalin-fixed paraffin embedded; CSF, cerebrospinal fluid; BAL, bronchoalveolar lavage; NGS, next generation sequence*.

## The Connections among Tumor Cytosol miRNAs, Fluid miRNAs, and Exosome miRNAs

**Figure 1:**
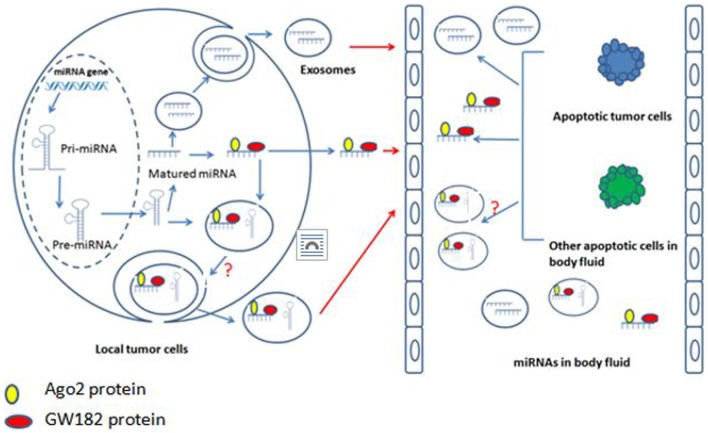
**The connection of tumor tissue miRNA, body fluid miRNA, and exosome miRNA**.

The maturation of miRNAs have been studied extensively and profoundly, and the well-accepted model is that the DNA coding miRNAs are transcribed into the primary miRNAs (pri-miRNAs), and then the pri-miRNAs are processed by Drosha (an RNase type III endonuclease) and specific cofactors to generate the precursor miRNAs (pre-miRNAs). Pre-miRNAs are transported from nucleus into cytoplasm where they undergo processing by Dicer and various cofactors to for mature miRNAs (56). But how the mature miRNAs enter into body fluids and what is the relationship between body fluid miRNAs and exosome miRNAs are still not clear. There are three possible ways for miRNAs to enter body fluids. First, the tumor cells in the primary location “secret” miRNAs, which combined with Ago2 and GW182 to enter body fluids (57, 58). Second, the miRNAs in the primary tumor cells are packaged into membranous vesicles (MVs) or exosomes and then enter into body fluids (59). However, it is still not clear if the incorporation of miRNAs into exosomes occurs at the pre-miRNA or mature miRNA level. Recently, Villarroya-Beltri et al. reported that exosomes contain mature miRNAs (60). Third, the tumor cells or other cells in body fluids directly release their cytosol miRNAs into body fluids after apoptosis (61) (Figure 1).

Currently, there is no consensus regarding the relationships between exosomal miRNAs and the body fluid miRNAs, especially the whole blood miRNAs. Several studies have indicated that the majority of miRNAs found in plasma and serum are present primarily outside the exosomes (62, 63), while other studies found that miRNAs in serum and saliva exist primarily inside the exosomes (54).

## Conclusion

Over the last decade, great progress has been made in the research of miRNAs and lung cancer. Several miRNAs with differential expression patterns in lung cancer tissues compared to normal tissues have been identified. Furthermore, aberrant expression patterns of miRNAs in lung cancer patients can not only be detected in tumor tissues but also in body fluids and extracellular organelles such as exosomes. All these studies give weight to the conclusion that miRNAs are promising biomarkers for diagnosis and prediction, as well as targets of potential therapeutics for lung cancer. Yet, before they can be effectively integrated into the field of clinical oncology, there are several issues that need to be addressed: (1) Using miRNA array analysis, it is easy to find numerous miRNA candidates whose expressions vary in lung cancer tissue compared to the normal tissue, or whose expressions vary in lung cancer patients body fluids and exosomes compared to the healthy persons. However, there is still no gold standard to evaluate meaningful candidates. It remains a challenge to increase the accuracy of the results from miRNA array analysis and validate meaningful candidates in an efficient manner. (2) Compared to tumor cytosol miRNAs, fluid miRNAs, and exosome miRNAs have attracted more attention as potential diagnostic markers for lung cancer, but there is no standard method for isolating these two kinds of miRNAs, and no reliable endogenous control for evaluation. (3) The quality control of the normal healthy tissue and fluid sample collections is also a key issue in the field, and obtaining a near perfect control at the nano-scale currently remains a major challenge. (4) The up-surging new information in this area brings more and more potential miRNA candidates for lung cancer, but how to interpret and integrate all the information into a network of knowledge for their clinical use as diagnostic and prognostic biomarkers and as potential therapeutic targets represents another attractive area for future investigation. Overall, the study of miRNAs offers a new and exciting angle for us to understand the molecular mechanisms of lung cancer biology. Further studies could provide more accurate biomarkers for both diagnosis and prediction, as well as improved strategies for lung cancer treatment.

## Author Contributions

All authors participated in conceiving the concept and writing the manuscript.

## Conflict of Interest Statement

The authors declare that the research was conducted in the absence of any commercial or financial relationships that could be construed as a potential conflict of interest.
